# Brilaroxazine lipogel displays antipsoriatic activity in imiquimod‐induced mouse model

**DOI:** 10.1111/srt.13606

**Published:** 2024-02-16

**Authors:** Laxminarayan Bhat, Seema R. Bhat, Arulprakash Ramakrishnan, Muthukumar Amirthalingam

**Affiliations:** ^1^ Reviva Pharmaceuticals, Inc. Cupertino California USA

**Keywords:** Baker scores, BALB/c, brilaroxazine, imiquimod‐induced, lipogel, PASI, proinflammatory cytokines, psoriasis

## Abstract

**Background:**

Dopamine (D) and serotonin (5‐HT) pathways contribute to psoriasis pathobiology. Disruptions incite increased inflammatory mediators, keratinocyte activation and deterioration, and worsening symptoms. Brilaroxazine (RP5063), which displays potent high binding affinity to D_2/3/4_ and 5‐HT_1A/2A/2B/7_ receptors and a moderate affinity to serotonin transporter (SERT), may affect the underlying psoriasis pathology.

**Methods:**

An imiquimod‐induced psoriatic mouse model (BALB/c) evaluated brilaroxazine's activity in a topical liposomal‐aqueous gel (Lipogel) formulation. Two of the three groups (n = 6 per) underwent induction with 5% imiquimod, and one group received topical brilaroxazine Lipogel (Days 1–11). Assessments included (1) Psoriasis Area and Severity Index (PASI) scores (Days 1–12), skin histology for Baker score based on H&E stained tissue (Day 12), and serum blood collection for serum cytokine analysis (Day 12). One‐way ANOVA followed by post hoc Dunnett's *t*‐test evaluated significance (*p* < 0.05).

**Results:**

Imiquimod‐induced animal Baker scores were higher versus Sham non‐induced control's results (*p* < 0.001). Brilaroxazine Lipogel had significantly (*p* = 0.003) lower Baker scores versus the induced Psoriasis group. Brilaroxazine PASI scores were lower (*p* = 0.03) versus the induced Psoriasis group (Days 3‐12), with the greatest effect in the last 3 days. The induced Psoriasis group showed higher Ki‐67 and TGF‐β levels versus non‐induced Sham controls (*p* = 0.001). The brilaroxazine Lipogel group displayed lower levels of these cytokines versus the induced Psoriasis group, Ki‐67 (*p* = 0.001) and TGF‐β (*p* = 0.008), and no difference in TNF‐α levels versus Sham non‐induced controls.

**Conclusion:**

Brilaroxazine Lipogel displayed significant activity in imiquimod‐induced psoriatic animals, offering a novel therapeutic strategy.

## 1 INTRODUCTION

Psoriasis is a systemic immune‐mediated, chronic‐residual dermal inflammatory disease with genetic components.^1^, ^2^ It presents as recurrent episodes of hyperkeratotic, erythematous plaques and silvery‐coated scales on the skin, symmetrically distributed to the extensor (knees, elbows), scalp, and lumbosacral (trunk) regions.^1^, ^2^ This common, incurable, chronic disease cycles with flares for weeks or months, then subsides periodically or remissions.^1^, ^2^ With a global prevalence of approximately 125 million, this condition manifests as phenotypically distinct subtypes, with plaque psoriasis accounting for over 80% of cases.^3^, ^4^ Mental illness (e.g., anxiety, depression, schizophrenia) is a major comorbidity in patients with psoriasis.^5^, ^6^, ^7^, ^8^, ^9^ Psoriasis patients are 1.5 times more likely to exhibit depressive symptoms and experience a higher prevalence of anxiety, schizophrenia, and suicidal ideation than those individuals who do not suffer from this condition.^7^ Its global burden and detrimental impact on patients’ health‐related quality of life (QOL) present a growing public health problem.^10^

While psoriasis's exact cause is unknown, predisposing factors (e.g., genetics, mutations, environmental factors, trauma, infection, wounds, drugs, and psychological or emotional stress) contribute.^2^ Psoriasis pathology involves an inflammatory skin response through extrinsic (e.g., environmental, physical, and lifestyle stressors) and intrinsic (e.g., mental and cardiometabolic stressors) factors.^2^ These stressors drive the activation of innate immunocytes (e.g., dendritic cells) and the modulation of adaptive immunocytes (e.g., T cells into Th1 cells), which subsequently release proinflammatory cytokines.^11^ Crosstalk between adaptive and innate immunity determines the clinical manifestations of psoriasis, with evidence showing more influence of adaptive immunity in chronic stable and mild disease. ^3^, ^11^ Whereas the actions of innate immunity influence severe, highly inflammatory, and/or pustular psoriasis.^3^, ^11^ These cytokines (e.g., tumor necrosis factor [TNF]‐α, interferon‐γ, and interleukins [IL]]‐12 and IL‐23) alter the immune system through a cascade of downstream abnormal differentiation and proliferation and leucocyte infiltration of lesioned keratinocytes.^3^ This inflammatory process generates rapid keratinocyte multiplication, followed by cellular movement from the epidermal basal layer to the upper layer of the epidermis occurs daily and the formation of thick dry patches or plaques.^3^

Current treatments include multiple modalities. Topical corticosteroids and vitamin D derivatives remain first‐line as monotherapy and complementary to systemic therapy despite the drawbacks of limited efficacy and long‐term side effects (e.g., tachyphylaxis, skin atrophy, adrenal suppression, and skin irritation).^3^, ^12^ Topical treatments can rapidly act and exert localized effects with minimal short‐term adverse events.^12^ Nonbiologic oral agents (e.g., methotrexate, apremilast, acitretin, or cyclosporine) offer more options for treating widespread inflammation; however, they are associated with significant toxicities (e.g., hepatotoxicity, nephrotoxicity, hypertension, dyslipidemia, malignancy, and teratogenicity).^3^, ^12^ Biologics (e.g., TNF and IL inhibitors) target specific immune response components.^12^ However, these agents’ use is limited due to the potential development of immunogenicity, serious infection and malignancy risk, parenteral administration, and affordability.^13^, ^14^

Undertreatment remains a concern, especially in severe cases and special populations, considering the complexity of managing a multisystem disease.^13^, ^14^ Hence, there is a need for new effective treatments with acceptable safety profiles and convenient administration routes.^15^, ^16^

Two relevant neurotransmitters play roles in the development of psoriasis. Serotonin (5‐HT) influences inflammation and immunity proliferation.^17^ These receptors are significantly altered in psoriatic skin, a potential target.^18^ Dopamine (D) plays a role in stimulating the production of proinflammatory cytokines and inciting the proliferation and differentiation of keratinocytes^19^, ^20^, ^21^ ^,^ and high D_2‐4_ receptor expression is present in psoriasis patients’ keratinocytes.^22^, ^23^, ^24^

Brilaroxazine (RP5063), a multimodal dopamine and 5‐HT receptor modulator, is a novel agent of interest. It displays a high binding affinity to D_2‐4_ and 5‐HT_1A_ receptors as partial agonists, 5‐HT_2A_ as a weak partial agonist or neutral antagonist, 5‐HT_2B/7_ as an antagonist, and a moderate affinity to serotonin transporter (SERT).^25^, ^26^, ^27^, ^28^, ^29^ Brilaroxazine has an established efficacy, safety, and pharmacokinetic profile from previous phase 1 and 2 studies in healthy volunteers and schizophrenia patients. ^25^, ^26^, ^27^, ^28^, ^29^ Also, preclinical work indicates that this agent inhibits the release of multiple proinflammatory cytokines.^25^, ^26^, ^30^ A new brilaroxazine liposomal‐gel (Lipogel) formulation has recently emerged, offering a novel topical option to explore.

This preclinical study examines whether brilaroxazine in this topical formulation displays efficacy in a psoriatic animal model. It assesses the research question: Does topical brilaroxazine in Lipogel produce greater efficacy, as compared to placebo‐liposome gel, in a 5% imiquimod‐induced psoriatic mouse model (BALB/c)? This paper's flow starts with study methods, segues to results, and closes with a discussion of these results relative to brilaroxazine's future role in psoriasis management.

## 2 METHODS

### 2.1 Procedures

The preclinical assessment of the brilaroxazine topical liposomal gel formulation (Lipogel) involved an imiquimod‐induced psoriatic mouse model (BALB/c) following guidance from Van Der Fits et al. (2009). This investigation received approval from the Institutional Animal Ethics Committee (IAEC) of SDM Centre for Research in Ayurveda and Allied Sciences (SDMCRA), Manipal, Karnataka, India, and maintained animals per protocol approval number SDMCRA/IAEC/KM‐P‐01.

The study utilized three groups (n = 6 per) of female BALB/c mice (8‐10 weeks old, weight + 5 gm): (1) Group 1 (Normal Control), (2) Group 2 (Psoriasis Control), and (3) Psoriasis + Brilaroxazine‐Lipogel‐ group. Groups 2 and 3 underwent psoriasis induction, using 5% imiquimod application to their shaved backs (3 cm X 3 cm area) the morning on Days 1–11. Group 3 received topical brilaroxazine–lipogel twice (1.5% w/w) daily, dosed topically after 3 h of imiquimod cream application and evening on Days 1–11.

At the end of Day 12, investigators sacrificed the animals and collected skin tissue from the tested area. They then performed histology preparation, staining, and assessment. Concurrently, they collected blood via a retro bulbar draw, separated the serum, and performed an enzyme‐linked immunosorbent assay (ELISA) analysis for cytokines.

### 2.2 Investigational drug‐lipogel delivery formulation

Brilaroxazine liposome preparation involved the lipid hydration method. The processes initially dissolved lipids and cholesterol, followed by brilaroxazine in chloroform and ethanol solvent. The next step involved drying the drug‐lipid solution at 45°C–50°C in a rotary evaporator using the vacuum to remove the solvent. Removal of all solvent led to a thin film in the round bottom flask, which then remained in a vacuum for 12–24 h to remove the traces in the thin lipid film completely. After this step, lipid film hydration involved incorporating a maltodextrin solution at 60°C.

Optical microscopic viewing at different magnifications (10x, 20x, and 40x) for confirmation of the prepared spherical‐shaped liposomal vesicles occurred throughout the hydration process. Hydrated lipid emulsion analysis via a dynamic light scattering method identified the drug‐lipid solution particle size (Z‐average 630.9 nm). High‐performance liquid chromatography (HPLC) showed a 96% drug content and 73% encapsulation efficiency. The final formulation composition was consistent with brilaroxazine (24.53%), lecithin (34.75%), cholesterol (2.97%), maltodextrin (37.74%), and purified water for lipid film hydration. After this step, the investigators used diverse lipogel formulation (0.25–1.5%) per need.

Using a Franz diffusion cell, Lipogel diffusion/permeation testing showed a flux of 12.02 μg/cm^2^/H and a permeation coefficient of 3.28 cm/h. The lipogel formulation showed a better drug release profile and the highest flux and permeation value in the in vitro diffusion study. This observation may be due to brilaroxazine's increased solubility in the liposomal gel and optimum particle size distribution in the formulation.

### 2.3 Assessments

Assessments included (1) Psoriasis Area and Severity Index (PASI) scores (observations on Days 1–12), (2) skin histology assessment using the Baker score based on Hematoxylin and Eosin (H&E) stained tissue (obtained on Day 12), (3) serum cytokine collection (collected via retro‐orbital puncture on Day 12), and (4) immunohistochemistry assessment for cytokine Ki‐67. PASI score calculation involved rating erythema, thickness, and scales on a four‐point scale (0 – None, 1 – Mild, 2 – Moderate, 3 – Severe, 4 – Very severe), followed by a summing of the scores for a total PASI score between 0 and 12.

For histology, tissue preparation involved fixed preserved skin tissues in the 10% formalin. Technicians sequentially prepared the tissue samples in blocks, cut them into thin 4‐micron sections using a microtome, and floated them in a water bath (50°C‐ 52°C). These individuals then situated the tissue samples in slides, placed them warmer at 55°C for 10 min, and stained the preparations using H&E. After drying the slide, the pathologist observed the prepared tissue under a microscope at 100 x and 400 x magnification to note changes in histology and signs of psoriasis toxicity on the skin. This individual then scored the stained tissue using the Baker scoring system on a scale ranging from 0 to 11: Keratin (Munro abscess 2.0, Hyperkeratosis 0.5, Parakeratosis 1.0), Epidermis (Thinning above papillae 0.5, Lengthening and clubbing of rete ridges 1.5, Acanthosis 0.5, Lack of granular layer 1.0), and Dermis (Lymphocytic infiltrate‐ Mild 0.5, Moderate 1.0, Marked 2.0, Papillary congestion 0.5).

Proinflammatory cytokine analysis involved serum Tumor Necrosis Factor‐alpha (TNF‐α), Ki‐67, and Transforming growth factor beta (TGF‐β). Technicians prepared and collected Day 12 blood samples per manufacturer's instructions for enzyme‐linked immunosorbent assay (ELISA) (TNF‐α, Medix Biochemica, France, Ki‐67 ‐ BT Lab Systems, USA, TGF‐β, Medix Biochemica, France) determinations, specified to each cytokine for presence and concentration. In addition, serum Ki‐67 concentration assessment involved ELISA assay. Investigators extrapolated all circulating levels from their respective standard curves. All tests were either colorimetric or fluorescence‐based.

For immunohistochemical staining (Ki‐67), after deparaffinization, antigen retrieval, and blocking 5 μm sample sections underwent overnight incubation with primary antibodies against Ki‐67 (Abcam, Burlingame, California, USA). The processes involved washing and incubating sections with an antibody enhancer (Cell Marque, Rocklin, California, USA) for 10 min at room temperature. Incubation of the sections occurred after washing. This process utilized horseradish peroxidase (HRP)‐conjugated antibodies (Cell Marque, USA) for 10 min at room temperature. The sections proceeded through washing and developing with 3,3′‐Diaminobenzidine (DAB, Cell Marque, Rocklin, California, USA) for 2 min at room temperature, followed by counterstaining with hematoxylin. Visualization and imaging of the sample section involved a digital camera (Axiocam 105, Jena, Germany) mounted on a phase contrast microscope (Zeiss, Jena, Germany) with Zen 2.3 software (Zeiss, Jena, Germany). Analysis of the mean optical density utilized ImageJ software (version 1.50i, National Institutes of Health, Bethesda, Maryland, USA).

### 2.4 Analysis

Data presentation involved mean ± SEM using GraphPad Prism software. The analysis utilized a one‐way ANOVA as a statistical test followed by Dunnett's multiple *t*‐test as a post hoc test. Statistical significance determination employed a *p* value < 0.05.

## 3 RESULTS

Evaluation of brilaroxazine Lipogel's activity comprises clinical observations using the PASI scoring, histological examination of samples from the animal's back using Baker scores with H&E stained tissue, and comparison of proinflammatory cytokine levels.

**FIGURE 1:**
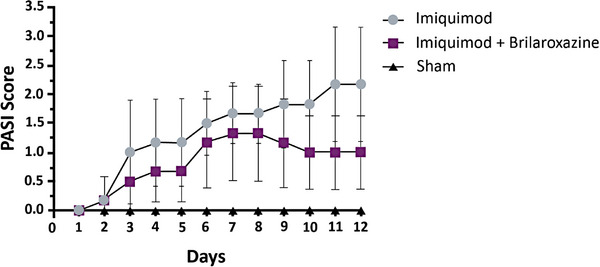
Comparative effects on PASI from Days 1 to 12 in the imiquimod‐induced psoriatic mouse model. PASI, Psoriasis Area and Severity Index between brilaroxazine Lipogel and induced Psoriasis group from Days 3 through 12 (*p* = 0.03).

The initial assessment involved daily clinical observations and PASI scoring. Figure 1 illustrates the composite PASI score for Days 1–12. The induced group (Psoriasis group) displayed higher PASI scores than the non‐induced control group (Sham control group) (*p* = 0.001). The differences in magnitude grow larger between these two groups starting from Day 3 out to Day 11. The brilaroxazine Lipogel group increased PASI scores starting on Day 3, peaking at Days 7 and 8, and fell to a plateau level on Days 10 through 12. The scores were higher in magnitude than the Sham control group but did not reach the same level as the Psoriasis group. Brilaroxazine Lipogel PASI scores were consistently lower than those in the induced Psoriasis group from Days 3 through 12 (*p* = 0.03). The maximum difference in magnitude appeared on Days 11 and 12.

**FIGURE 2:**
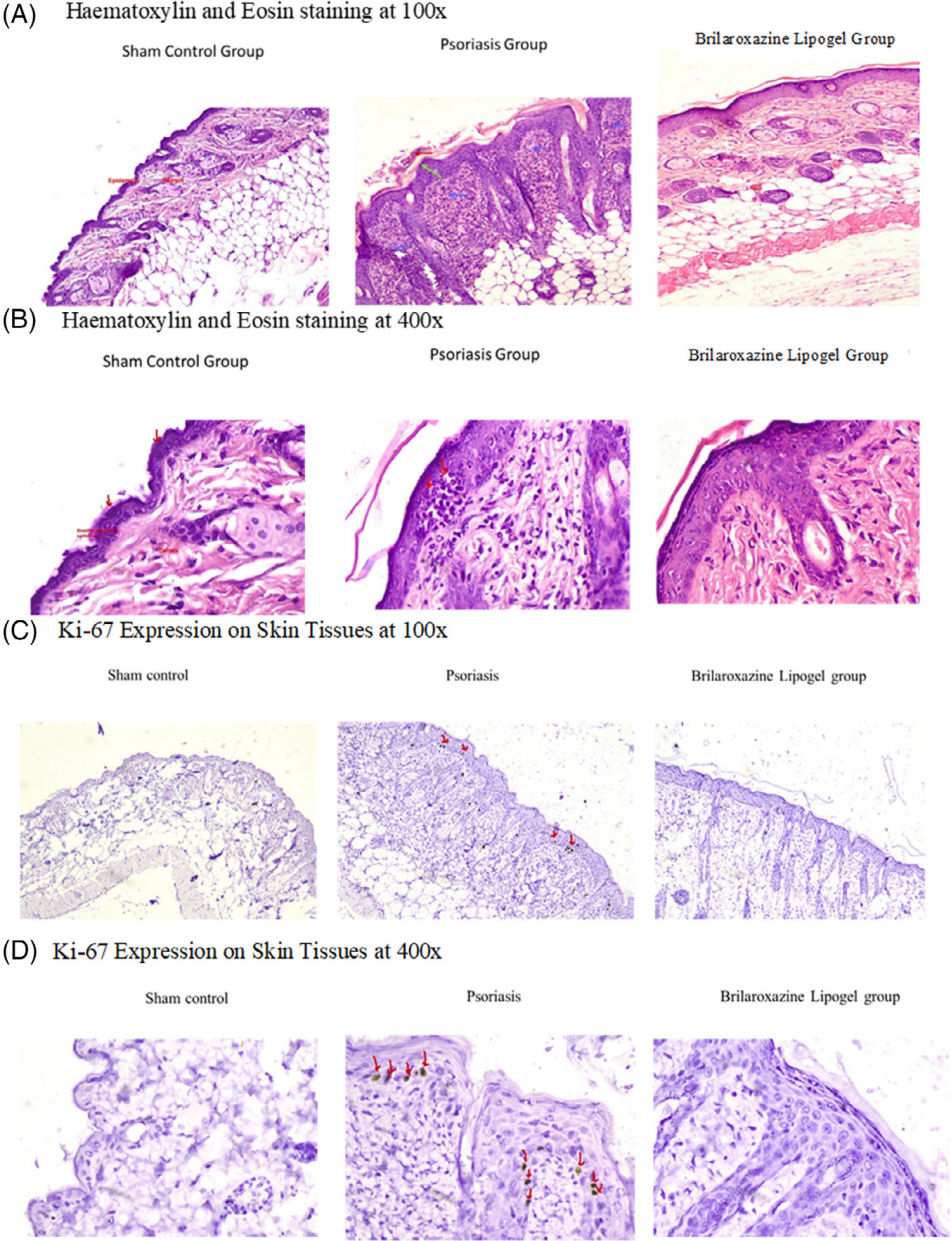
Hematoxylin and Eosin staining of skin histology studies at (A) 100x and (B) 400x magnification and Ki‐67 expression on skin tissues at (C) 100x and (d) 400x magnification for Sham control, Psoriasis, and Brilaroxaine Lipogel groups in the Imiquimod‐Induced Psoriatic mouse model. The red arrows in c and d indicate Ki‐67 positive cells.

**FIGURE 3:**
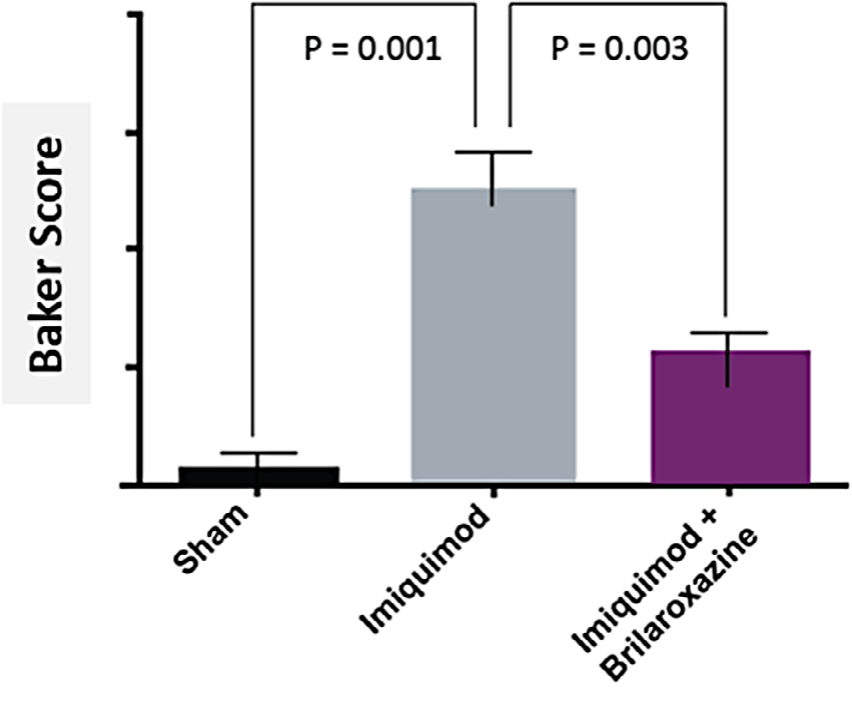
Baker scores for Sham control, psoriasis, and brilaroxazine lipogel groups in the imiquimod‐induced psoriatic mouse model.

Histology evaluation includes direct observation at 100 x and 400 x magnification. Table 1 and Figure 2A,B provide histological observations and H&E staining at both magnifications. Differences appear between the Sham control with the induced Psoriasis and brilaroxazine Lipogel groups and between the latter two groups. Baker score comparisons (Figure 3) reflect significant effects for the Sham control (*p* = 0.001) and the Brilaroxazine Lipogel (*p* = 0.003) groups, versus the Psoriasis cohort. Such observation highlights the treatment effect of Brilaroxazine Lipogel.

**TABLE 1:** Histological observations at 100x and 400x .

100x magnification	
Group	Observations
Sham control group	Normal epidermis (1‐3 layers), no inflammatory infiltration
Psoriasis control group	Increased epithelial layers 3–7 layers (green), increased keratinization, Munro's abscess (red arrow), severe inflammatory (blue) infiltration
Brilaroxazine Lipogel	Reduced epithelial thickness (3‐4 layers), reduced inflammatory infiltration, absence of parakeratinization, absence of Munro's abscess

400x Magnification	
Group	Observations
Sham control group	Epithelium 1–3 layers, no inflammatory infiltration, very little keratin in stratum corneum
Psoriasis control group	Severe acute and chronic inflammatory infiltration, Kogoj pustule
Brilaroxazine Lipogel	Reduced epithelial thickness, reduced inflammatory infiltration

**FIGURE 4:**
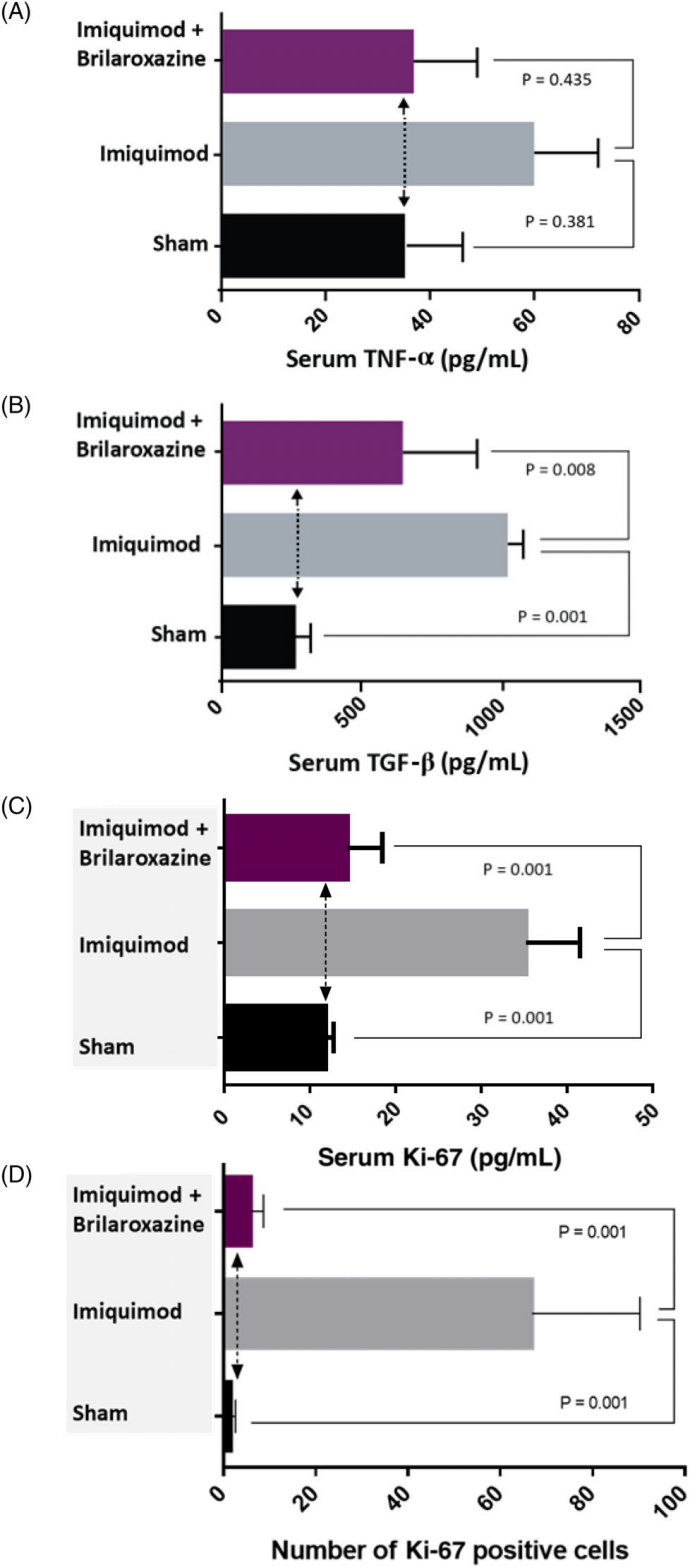
Serum proinflammatory cytokine levels (A) TNF‐α, (B) TGF‐β, (C) Ki‐67, and (D) Skin Ki‐67 levels for Sham Control, Psoriasis, and Brilaroxazine Lipogel groups in the imiquimod‐induced psoriatic mouse model. TGF, transforming growth factor; TNF, tumor necrosis factor.

Cytokine assessment involves serum TNF‐α, Ki‐67 and TGF‐β (Figure 4A,B and C) effects, comparing Sham, Psoriasis, and brilaroxazine Lipogel groups. The Psoriasis group reflects significantly higher Ki‐67 and TGF‐β levels versus non‐induced Sham controls (*p* = 0.001). The brilaroxazine Lipogel group displays significantly lower TGF‐β (*p*=0.008) and Ki‐67 (*p*=0.001) than Psoriasis group. Serum TNF‐α levels for the Psoriasis group are higher in magnitude but insignificant (*p* = 0.381). The brilaroxazine Lipogel TNF‐α levels appear lower in magnitude than the Psoriasis group (*p* = 0.435) and similar to the Sham non‐induced controls (NS).

Figures 2C,D and 4D provide results for the skin Ki‐67 immunohistochemistry analysis (number of positive cells). The Psoriasis group displays a significantly (*p* = 0.001) higher number of Ki‐67 positive cells than the sham control group. The brilaroxazine lipogel displayed treatment efficacy with significantly fewer Ki‐67 positive cells (*p* = 0.001). Additionally, brilaroxazine lipogel showed significant reduction in serum Ki‐67 (*p* = 0.001) in ELISA assay (Figure 4C).

## 4 DISCUSSION

This preclinical study represents the initial proof‐of‐concept for brilaroxazine's activity in a liposomal gel formulation. The contributions were fourfold. First, it provides the first evidence relative to the brilaroxazine Lipogel's activity in animals. Second, it highlights the viability of this topical drug‐delivery system as applied to psoriasis. Third, it offers proof‐of‐concept to support dopamine and serotonin receptors as viable psoriasis targets and an initial glimpse at the histological and cytokine changes indicating potential effects on inflammation and fibrosis. Finally, the results using the imiquimod‐induced psoriatic mouse model (BALB/c), a standard for psoriasis preclinical work, support the viability of this approach to screen pharmacologic activity. Notable is that the Baker's scores for the imiquimod‐induced treated and non‐treated animal groups were higher than the Sham non‐induced control's results (*p* < 0.001), indicating the model's viability.

Accordingly, it extends other researchers’ prior works using it for pharmacologic activity screening in this condition.^32^, ^33^, ^34^, ^35^ A popular, widely utilized model to study psoriasis‐like inflammation in mice is the recurrent application of topical imiquimod, a Toll‐like receptor 7/8 agonist.^36^ Commercially available, 5% imiquimod cream is applied daily to mice's backs and/or ears for 5–7 days to induce an inflammatory response reminiscent of human psoriasis.^37^ Important hallmarks of imiquimod‐induced psoriasis are the infiltration of plasmacytoid dendritic cells and the T_H_17‐dominated immune response with IL‐23 as a key driver.^38^ The following assessments, PASI score, ear thickness measurements, histological staining, splenic involvement, and the role of inflammatory cytokines have been very well established in this model.^31^, ^39^, ^40^ This model has limitations regarding differing immunopathology and limited similarity to immune and inflammation‐associated gene expression in psoriatic skin and imiquimod‐induced inflammation.^41^, ^42^

Concerning proof‐of‐concept, this study evaluated multiple psoriatic measures in the in vivo preclinical setting. These included observational, such as the PASI, histological (including the H&E stain, observations, and Baker scores, and proinflammatory cytokine levels). The most notable findings were brilaroxazine Lipogel's significant effects on psoriatic skin histology and Baker scores, compared with the Psoriasis control. These findings suggested that brilaroxazine Lipogel mitigated some of the underlying psoriasis pathology involving inflammation and fibrosis by first its action on 5‐HT as a target and second the drug's ability to effectively penetrate the affected tissue in sufficient concentration.

Brilaroxazine Lipogel's effect on the proinflammatory cytokines, TNF‐α and TGF‐β, was also of interest, indicating its anti‐inflammatory effect. TGF‐β were significant compared with the Psoriasis group. The TNF‐α did not show a significant difference versus the Psoriasis control group, but brilaroxazine Lipogel's effect on this cytokine showed that it produced the same serum level as the Sham control.

It is important to unpack this nonsignificant finding. Potential explanations include the small sample size due to study animal ethical considerations and that cytokines levels were not the primary endpoint for consideration. Also, differences in effect for TNF‐α might have emerged due to sample size considerations and time point of sampling (Day 12). This cytokine might require a longer treatment to see differences between the brilaroxazine Lipogel and Psoriasis groups. Other animal studies for inflammatory conditions have documented brilaroxazine's effects on proinflammatory cytokines, including significant effects (*p* < 0.05) on serum TNF‐α, IL‐1β, IL‐6, and LT‐B4 levels.^25^, ^26^

Ki‐67 immunohistochemistry analysis provides significant observations reflecting both the development and attenuation of the psoriatic pathologic process. Ki‐67 is an established marker for cell proliferation.^43^, ^44^, ^45^ It is highly expressed in psoriasis and correlates with the clinical severity of this condition.^44^ Most of the cell cycle's stages contain this marker.^43^ Compared to healthy, lesion‐free skin, psoriasis lesions express Ki‐67 more intensely.^46^ The Psoriasis group significantly increased Ki‐67 positive cells (*p* = 0.001) versus Sham in this study. This group developed the psoriatic pathology, demonstrating the model's feasibility and viability. The brilaroxazine Lipogel group shows significantly lower Ki‐67 positive cells than the Psoriasis‐induced cohort (*p* = 0.001), indicating that this treatment strategy mitigates the psoriasis cellular proliferation process.

Next, brilaroxazine Lipogel's PASI scores were significantly lower from Days 3 to 12 (*p* = 0.03). Most interesting was the earlier time of peak scores, and the most extensive differences existed on Days 10–12. Such considerations are essential to consider the effect in this model because inflammatory and fibrotic conditions can take time to express themselves fully and might require more time to see the most extensive effect to express in the disease state, particularly in this model.^37^

These effects appear to reflect the modulation of dermal 5‐HT and D receptor actions. Increasing evidence suggests that 5‐HT regulates inflammation and immunity in disease processes such as psoriasis, particularly 5‐HT_2B/7_ receptors.^17^ It acts as a strong chemoattractant to recruit innate immunocytes to inflammation sites, modulating cytokine and chemokine production and promoting cell activation and proliferation.^17^ Several 5‐HT receptors, including 5‐HT_1A/2A/2B/7_ and SERT, have been found in numerous immunocytes and non‐immunocytes (e.g., keratinocytes).^47^ Receptor expression appears significantly altered in psoriatic skin at multiple dermal layers, which, in addition to the involvement of systemic cytokines inflammatory mediators (e.g., TNF, IL), contribute to this condition's pathogenesis ^18^, ^48^, ^49^, ^50^ Similarly, increasing evidence suggests D regulates the proliferation and differentiation of keratinocytes, modulating the immune system, particularly via the D_2‐4_ receptors.^22^, ^24^ Studies indicate that D agonists stimulate the production of IL‐6 and IL‐8, which play a role in the proliferation and differentiation of epidermal cells.^19^, ^21^ Other works indicate higher receptor expression of D_2_‐like receptors (D_2‐4_) and IL‐8 psoriatic patient keratinocyte cells and contribute to the autoimmune processes in these patients.^22^, ^24^

Brilaroxazine appears to influence the psoriasis pathobiology process through effects on D_2‐4_ receptors, 5‐HT_1A/2A/2B/7_ receptors, and SERT.^25^, ^26^, ^29^ Most notable is its mitigation of proinflammatory cytokine levels in pulmonary hypertension^25^, ^26^ and anti‐fibrotic effects, as evidenced by its reduced collagen levels in idiopathic pulmonary^47^ fibrosis.^30^ Further, brilaroxazine Lipogel's topical, semisolid delivery system provides targeted, direct contact with the plaque target area, which can make a difference in getting adequate levels in the affected skin. Such administration leads to direct drug penetration, mediating 5‐HT's actions on multiple dermal layers to control the disease process.^51^ Lastly, it reduces systemic exposure and side effects associated with oral delivery.^51^

Such brilaroxazine Lipogel delivery characteristics offer potential for psoriasis, but this promise needs translation from animals to the clinic. Currently, brilaroxazine's clinical development program involves schizophrenia as a lead indication in clinical phase 3 studies for its oral formulation with plans to further expand the portfolio in bipolar disorder, major depression disorder (MDD), attention deficit hyperactivity disorder (ADHD), pulmonary arterial hypertension (PAH), and idiopathic pulmonary hypertension (IPF) as rare disease indications^52^, ^53^, ^54^ These directions may offer a connection to psoriasis. First, psoriasis appears relevant as there appears to be a relationship between patients with mental illness with psoriasis, which may occur in as high as 36% of such patients.^5^, ^6^, ^7^, ^8^, ^9^ Next, psoriasis is a condition that significantly impairs the psychosocial functioning of patients, decreasing their quality of life and, in extreme cases, causing depression, anxiety, or even suicidal ideation.^55^ Another key consideration is that brilaroxazine's effects appear to include ameliorating neuroinflammation due to proinflammatory cytokines and chemokines (e.g., C‐reactive protein, IL‐1β, IL‐2, IL‐4, IL‐6, TNα, INFγ) mediated by 5‐HT_1A/2A/2B/7_.^30^, ^56^, ^57^, ^58^ Finally, brilaroxazine has shown effects on proinflammatory cytokines in animal models of PAH^25^, ^26^ and IPF.^30^

As with any early study, some limits do exist. In this case, the most notable would be the number of animals per group and the duration of treatment. Such considerations would help to tease out statistical significance, particularly related to TNF‐α level comparisons between brilaroxazine Lipogel and the Psoriasis groups, particularly with the Sham control cohort. Animal ethical considerations and protocol standardization might explain this consideration. The study included another proinflammatory cytokine, TGF‐β, which showed significant differences between the Psoriasis group versus Sham and brilaroxazine Lipogel. Such observations reflect the treatment effect on the elevated cytokine levels in the induced group. Finally, the potential differences in sensitivity for the mode of action of brilaroxazine between humans and animals must be considered when calculating the starting dose in the clinical setting.

Hence, the future agenda to extend this work should include these considerations. Such efforts would aid in fully elucidating the underlying mechanisms directly and indirectly related to brilaroxazine's actions on 5‐HT and D receptors, proinflammatory cytokines, and the development of fibrosis. These data would reinforce this initial, promising proof‐of‐concept work. Additionally, to move to the clinic, brilaroxazine will need to complete its preclinical package to include relevant safety, toxicity (short and long‐term due to the chronic nature of psoriasis management), and pharmacokinetic studies, and Chemistry, Manufacturing and Control data on this topical formulation. After completing this preclinical work, phase 1 and 2 studies can follow to translate the initial findings from this work fully.

## 5 CONCLUSION

Psoriasis, a condition involving inflammation and fibrosis in its pathology, affects 125 million globally.^3^, ^4^ Evidence suggests that 5‐HT and D contribute to psoriasis's pathophysiology, particularly 5‐HT_2B/7_ and D_2‐4_ receptors, respectively.^17^, ^19^, ^21^ Brilaroxazine is a novel modulator of 5‐HT and D. It possesses a high binding affinity to D_2‐4_ and 5‐HT_1A/2A_ as a partial agonist and 5‐HT_2B/7_ as an antagonist, and a moderate affinity to SERT.^25^, ^26^, ^29^

This preclinical study represents the first evaluation of brilaroxazine's activity in a topical Lipogel formulation for psoriasis. It offers an initial proof‐of‐concept in the condition. This investigation involved the imiquimod‐induced psoriatic mouse model (BALB/c), a standard for anti‐psoriatic drug activity. Baker scores for imiquimod‐induced treated and non‐treated animal groups were higher than the Sham non‐induced control's results (*p* < 0.001),^31^ indicating the model's viability. Histological findings within the brilaroxazine Lipogel group showed notable differences in observed tissue changes via H&E staining and a statistically significant difference in Baker scores compared with the Psoriasis group (*p* = 0.003). These findings indicate a treatment effect. The brilaroxazine Lipogel group also displayed lower PASI scores than the Psoriasis control from Days 3 to 12 (*p* = 0.03). Finally, the brilaroxazine Lipogel group had lower serum and skin Ki‐67 levels (*p* = 0.001), and lower serum TGF‐β levels (*p* = 0.008) than those in the induced Psoriasis group. It showed no difference in TNF‐α levels versus the Sham non‐induced controls. Such findings suggest an observable clinical effect and an impact on proinflammatory cytokines that may play a role in the pathogenesis of psoriasis.

Considering the challenges with current psoriatic treatments, brilaroxazine in a lipogel formulation offers a new target and novel delivery system. This approach represents a clinically attractive option to pursue further human investigations. Further clinical work can help translate these findings and confirm this initial animal proof‐of‐concept in the clinical setting.

## CONFLICT OF INTEREST STATEMENT

Laxminarayan Bhat, Ph.D., Seema R Bhat, M.Sc., and Arulprakash Ramakrishnan, MPharm are employees and Muthukumar Amirthalingam, Ph.D. is a consultant of Reviva Pharmaceuticals, Inc.

## Data Availability

The data that support the findings of this study are available on request from the corresponding author. The data are not publicly available due to privacy or ethical restrictions.
