# Correction to “Brilaroxazine Lipogel Displays Antipsoriatic Activity in Imiquimod‐Induced Mouse Model”

**DOI:** 10.1111/srt.70329

**Published:** 2026-01-14

L. Bhat, S. R. Bhat, A. Ramakrishnan, and M. Amirthalingam, “Brilaroxazine Lipogel Displays Antipsoriatic Activity in Imiquimod‐Induced Mouse Model,” *Skin Research and Technology* 30, no. 2 (2024): e13606, https://doi.org/10.1111/srt.13606.

In Figure 3 of the published article, the error bars for SEM are incorrectly displayed and the units on the y‐axis were not presented. The correct Figure 3 addressing these issues is presented below.

**FIGURE 3:**
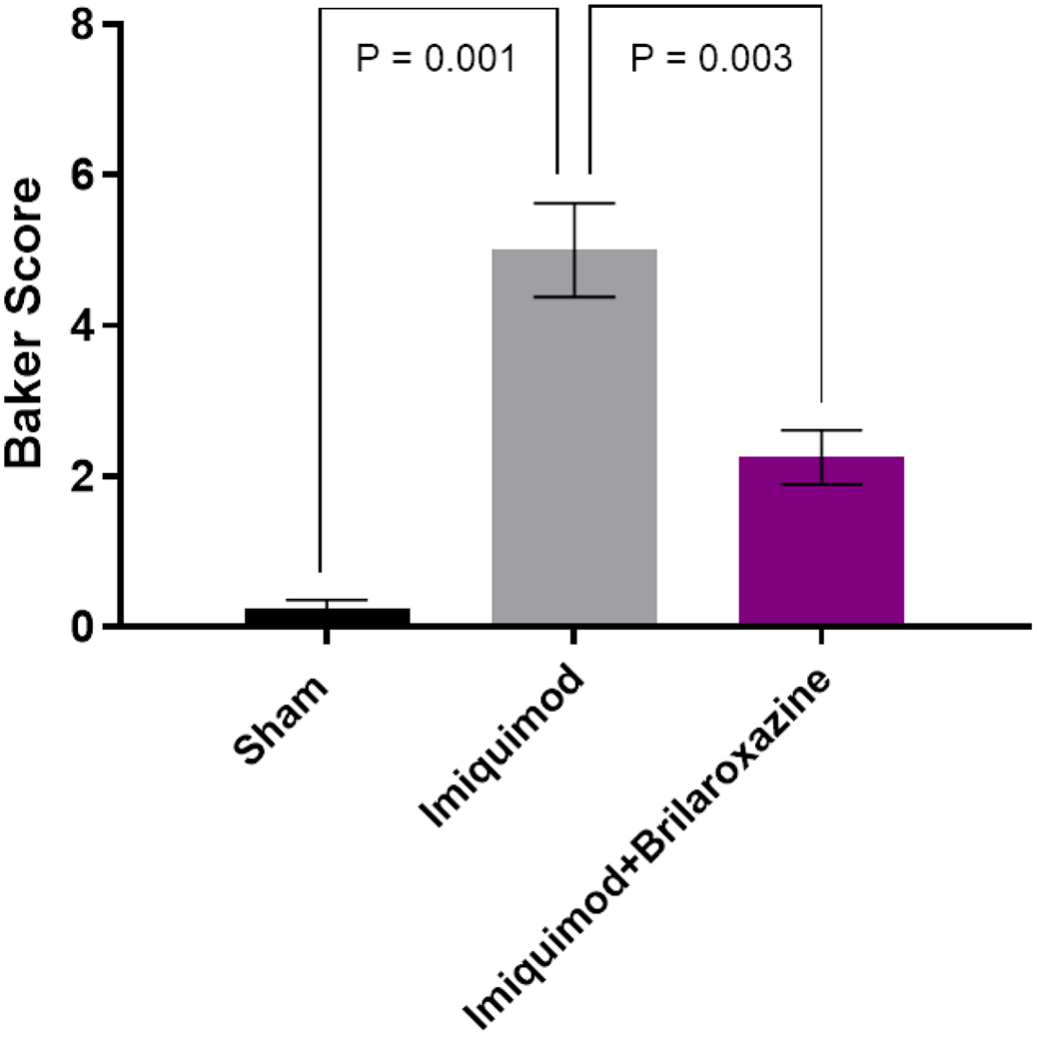
Corrected.

Furthermore, the “Acknowledgments” section is corrected from:

John M. York, Pharm.D., M.B.A., Akita Biomedical, Inc. provided editorial support. Reviva Pharmaceuticals, Inc. funded this effort.

To:

John M. York, Pharm.D., M.B.A., Akita Biomedical, Inc. provided editorial support. Reviva Pharmaceuticals, Inc. funded this effort. The Shri Dharmasthala Manjunatheshwara (SDM) Centre for Research in Ayurveda & Allied Sciences (SDMCRA), SDM College of Ayurveda Campus, Manipal, Karnataka, India, performed all in vivo procedures under Institutional Animal Ethics Committee approval. Arul Amuthan, PhD, the lead investigator at SDMCRA, oversaw the animal study, and research staff under his supervision conducted animal handling, treatment administration, and histological scoring. They procured the animals used in this study from Bangalore, Karnataka.

The authors apologize for these oversights and for the inconvenience they may have caused.

